# Obstacles and opportunities for monitoring ethnicity-based inequalities in maternal health care: Lessons from Mexico

**DOI:** 10.1371/journal.pone.0217557

**Published:** 2019-05-31

**Authors:** Nancy Armenta-Paulino, María Sandín Vázquez, Francisco Bolúmar

**Affiliations:** Public Health Unit, Faculty of Medicine, University of Alcalá, Alcalá de Henares, Spain; Newcastle University, UNITED KINGDOM

## Abstract

**Background:**

Monitoring and reducing inequalities in health care has become more relevant since the adoption of the Sustainable Development Goals (SDGs). The SDGs bring an opportunity to put the assessment of inequalities by ethnicity on the agenda of decision-makers. The objective of this qualitative study is to know how current monitoring is carried out and to identify what factors influence the process in order to incorporate indicators that allow the evaluation of inequalities by ethnicity.

**Methods:**

We conducted 17 semi-structured interviews with key informants from the health ministry, monitoring observatories, research centers, and international organizations, involved in maternal health care monitoring in Mexico. Our analysis was interpretative-phenomenological and focused on examining experiences about monitoring maternal health care in order to achieve a full picture of the current context in which it takes place and the factors that influence it.

**Results:**

The obstacles and opportunities pointed out from the participants emerge from the limitations or advantages associated with the accuracy of evaluation, availability of information and resources, and effective management and decision-making. Technicians, coordinators, researchers or decision-makers are not only aware of the inequalities but also of its importance. However, this does not lead to political decisions permitting an indicator to be developed for monitoring it. As for opportunities, the role of international organizations and their links with the countries is crucial to carry out monitoring, due to political and technical support.

**Conclusions:**

The success of a monitoring system to help decision-makers reduce inequalities in health care depends not only on accurate evaluations but also on the context in which it is implemented. Understanding the operation, obstacles and opportunities for monitoring could be a key issue if the countries want to advance towards assessing inequalities and reducing health inequities with the aid of concrete policies and initiatives.

## Introduction

Indigenous women are among the most vulnerable groups in Latin America. They live in contexts where different social determinants such as poverty, low education, gender roles and cultural factors are combined.[1] Indigenous women experience significantly worse maternal health outcomes (including high risk of maternal mortality) and have more limited access to health services than majority populations. [2] Besides, historically, indigenous populations have not been made visible in the statistics, which could hide deep inequalities concerning other populations groups that would require a prompt response from governments and health systems. [3]

In this context, the monitoring of inequalities by ethnicity is useful and necessary to identify where there are differences. Regular health inequality monitoring can help to determine the impact of policies, programs, and practices, and to inform changes required to reduce inequality. The Government, the Ministry of Health and other stakeholders, could focus research in these areas to determine the cause of the problems and improve the health situation in these populations. [4,5]

Since the Sustainable Development Goals (SDGs) were established, monitoring health inequalities is gaining attention as a political priority. The SDGs agenda provides a major impetus for establishing or strengthening systems for monitoring health inequalities and calls for the production of disaggregate data. Further, SDGs call to continue the efforts to reduce maternal mortality and inequalities within and between countries. [6–8] Therefore, now is the time to assess ethnicity-based differences.

Some research has already shown how to estimate ethnicity-based inequalities in maternal health care. These studies give information about indicators and approaches for identifying the indigenous population using data obtained from national surveys. [9–13] In addition, international agencies or initiatives have published manuals and given technical assistance to support countries in building capacity for integrating health inequality monitoring into their health information systems. [4,5]

Nevertheless, is that enough to allow the monitoring of inequalities by ethnicity? The answer is not so easy, because it is not carried out in isolation and goes beyond the estimation of indicators. Monitoring involves the cooperation and interaction of various actors if it is to be done efficiently and be of use in decision-making. It is a process which takes place in a social, political and scientific context which brings together numerous institutions and individuals, all with their interests which impact on issues like indicator selection, data sources, methods, and leadership. [12,14]

Therefore, now is the time to take a hard look at the political and technical obstacles that might be forthcoming if we want to move forward to monitoring health inequalities. For this purpose, we need to fully understand the current operation of the maternal health care monitoring and the factors that impinge on it, from the perspective of the actors involved in it.

The main objective of the study is to describe how the monitoring of maternal health care is carried out in Mexico. Through the perspective of key professionals involved in the monitoring from, we seek to discover what factors influence the monitoring process and to explore the feasibility of incorporating an indicator that measures inequalities by ethnicity.

## Methods

### Sample and participants

In the process of maternal health care monitoring different types of professionals are involved with specific activities. Some are familiar with information aspects while others with how to use the results. Therefore, we used stratified purposive sampling to include this diverse kind of informants that could provide detailed and relevant information about maternal health care monitoring in Mexico. [15–17] We considered different interest profiles with specific discourses such as decision-makers, coordinators, technicians, and researchers from the Health Ministry, monitoring observatories, research centers, and international organizations. The purpose of using this sampling was to reflect the diversity within a population rather than looking for statistical representativeness or generalizability. [18]

**Fig 1:**
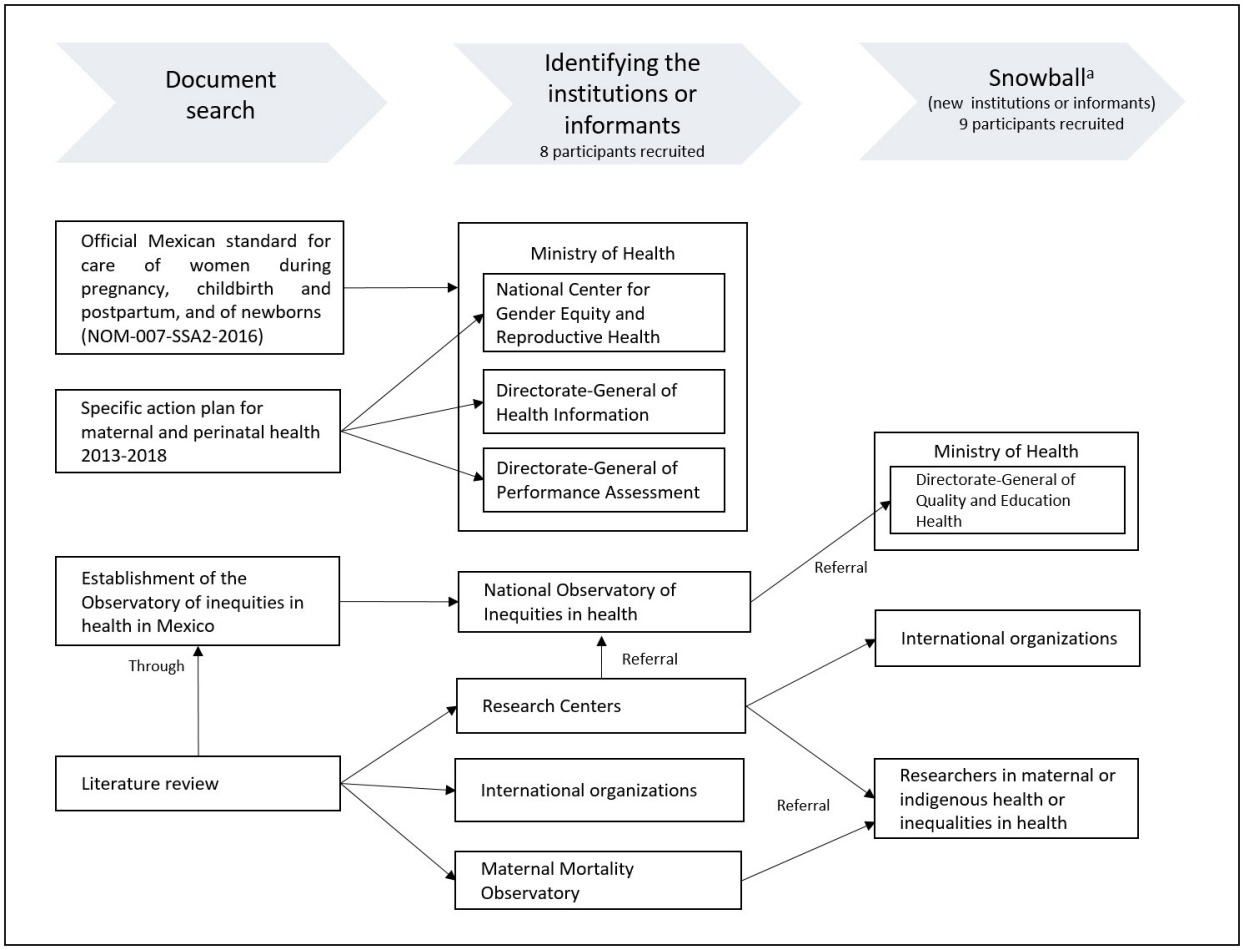
Sampling procedure and main institutions involved in the monitoring of maternal health in Mexico. Source: Own elaboration using information from the author’s analysis of systematic document search. Through this strategy, we also identified new areas or informants in the same organizations.

We began contacting and recruiting participants through a systematic document search to identify the main actors involved in monitoring. We identified the key informants within each institution or area, explained to them the study aims and invited them to participate. Finally, we applied the snowball strategy, and the participants were asked to indicate other institution or actors who might also be associated with the monitoring (Fig 1). [16,17]

The resulting sample consisted of 17 participants from different institutions and profiles as described in Table 1.

**Table 1:** Main actors and participant’s profiles.

**Actors**	
Sector or institution	Responsibilities and activities [11,19–25]
Ministry of Health	It has primary responsibility for monitoring the indicators, within it, the departments involved include those entrusted with data collection, health services performance assessment, and policy-making in the fields of sexual and reproductive health
National organizations	The Maternal Mortality Observatory is responsible for measuring and monitoring mortality at the national or regional level, and public policies aimed at reducing it. The National Observatory of Inequalities in Health focuses on identifying inequalities in health care to reorient public policies to reduce disparities.
Research field	Various groups of research tackle issues of maternal health from multidisciplinary perspectives and have measured inequalities across different population groups, taking some account of ethnicity in the process. Some researchers collaborate or form part of the monitoring observatories
International Organizations	In the sphere of international cooperation, different agencies and initiatives are at work in Mexico, where they are developing a variety of tools to carry out monitoring or assess inequalities. They, also, do advocacy to implement the monitoring of disparities. Some collaborate at the national level with the health ministry, others with local health service providers; they are also linked to research teams and monitoring observatories
**Participants**^a^^b^	
Profile	Description
Technicians (n = 4)	Responsible for estimating indicators or generating information for monitoring
Coordinators (n = 4)	Area directors, regional or local coordinators responsible for information management or monitoring indicators. They are the link between the technicians and the decision-makers and coordinate the activities of estimation or generation of data and deliver the results to the final users
Decision-makers (n = 5)	General management or representatives of initiatives or projects. They could be the final users who analyze the results of the estimates and share it or report it to other political actors
Researchers (n = 4)	Researchers with experience in methodologies for data analysis in maternal or indigenous health or its inequalities

Sources: Own elaboration using information from the author’s analysis of systematic document search.

^a^ The sample includes at least one member of the first three profiles from the health ministry, national and international organizations. The researchers are linked to an academic institution, and some of them collaborate with the monitoring observatories.

^b^ Other participants characteristics are available in S1 Table.

The sample size was determined considering theoretical saturation and the relevance and novelty of the findings. [15,17] Concerning the first, the information gathered was regarded as sufficient when there was no longer any variety of ideas or new elements that enriched or took the subject of study any further. Priority was also given to information that contributed to the goals of our research or had something new to lend to the discussion.

### Data collection procedure

Semi-structured interviews were conducted with a scheme covering: the respondent’s experience and role in the process, monitoring indicators or estimates used, information sources, monitoring problems and needs, interest groups and vulnerable groups, and aspects of strategic management.

We conducted the interviews between January and March 2018. Each interview was recorded and lasted an average of 60 minutes. We transcribed and coded the audios to preserve the respondents’ anonymity. All participants were informed of the purpose of the study, and once they agreed to participate, they completed written consent forms. The University of Alcalá’s Ethics Committee approved the study’s procedures (resolution no. CEID/HU/2018/38).

### Methodological approach and analysis

We chose a qualitative approach to explore the key professional perceptions of maternal health care monitoring. The qualitative research enables an understanding from the perspective of the professionals involved on how the current monitoring happens or which factors affect it. [26]

The main approach of the study is descriptive. However, we considered the interpretative-phenomenological analysis (IPA) as an adequate complement to exploring in detail how participants experience their work in the monitoring. IPA builds a detailed case by case interpretation of the phenomena in question, allowing us to take account of the individual differences in experiences as well as what is shared by all participants. Our study included different types of professionals (researchers, technicians, coordinators or decision- makers) because they would have different versions of the same event (maternal health care monitoring). Hence, the IPA was used to understand the content and meaning of the information collected from the perspective of the participants. [26,27,28]

Our analysis was based upon a detailed case exploration, and we reviewed individually every transcript before moving to the next case. Each transcript was read again and again to select the most significant aspects mentioned by the participants in their responses and to identify similarities, contradictions, differences or additional input. We highlighted phrases and paragraphs of interest and annotated all possible relevant topics. Subsequently, the initial coding was transformed into emerging themes and looked for possible connections between them. Themes were clustered together, and some emerged as superordinate concepts. The first case’ themes were used as a reference to the analysis of the following cases, but also incorporated the new emerging themes found through the other transcripts. [28] Two researchers defined the general issues and the main themes as a mean to ensure the quality and rigor of the analysis of responses.

Tables 2 and 3 show the final list of themes and subthemes that arose as a function of the study’s goals, the wealth and quality of the information it provided, and their contribution to understanding and exploring more deeply the subject in hand. Five themes were identified in relation to obstacles and opportunities for maternal health care monitoring. These concern evaluation, information, resources, management, and decision-making.

**Table 2:** Themes and sub-themes identified as obstacles to monitoring maternal health care.

Themes	Sub-themes	Description and example quote
Evaluation	Information systems	Subgroups cannot be analyzed. *“You have the information and you can calculate the indicator*, *but if you’re interested in a particular group*, *you can’t always evaluate it…”* [Health sector technician]
	Methodological approach	Processes involve many elements; it is difficult to determine the denominators in the indicators, or the evaluations cannot be reproduced. *“The point about the data the services generate is that you have to look carefully at the denominators they use*. *Because they usually correspond to the response that the system can give and not necessarily to the whole population it should coverage”* [Decision-maker, international body]
Information	Quality information system	Low quality; inconsistent and deficient capture at irregular intervals. *“When you use administrative sources there are great challenges in terms of standardizing the capture of these data*. *Because you go to the health departments where this kind of information is captured and the person who collects it is not necessarily as qualified in one place as in another”* [Researcher]
	Delays information system	The data supplied by the system are delayed, chiefly on account of the installation of a new system. *“I filled in a form asking for an update on over 24 results indicators and have received no reply*. *I’ve already filled in three more making the same request*.*”* [Health sector coordinator]
	Surveys	Unrepresentative at local level or for certain groups. No guarantee that they will be carried out once again. *“Only one round of this survey included these questions because they’re always making small changes to the surveys*. *We could only measure it using this round*.*”* [Researcher]
Resources	Budget/Funding	Difficult to obtain funding for this kind of project or no budget allocated to carry it out. *“We’ve looked for funding for this kind of analysis*, *but there has been no interest*. *I think they’re under the mistaken impression that [health inequity monitoring] is all very obvious and that there’s no need for resources to do it”* [Researcher]
	Time	Overwork limits the time available for monitoring. *“I have to monitor the indicators*, *I have to represent [to the sector and my area] at different committees*, *collaborate in audits*, *etc*. *So yes*, *the workload is a bit of a strain*.*”* [Health sector coordinator]
	Human resources	Shortage of staff or collaborators to perform monitoring. *“All the monitoring was my work because in [the institution] we were a very small group of three or four and each one was centered on his or her project”* [Observatory technician]
Management	Bureaucracy	The bureaucratic system hampers decision-making; or the paper-work for performing monitoring. *“There are challenges regarding working with [the local health sector] because of the bureaucratic system*, *which is what it is and we cannot change it*. *Some people want to shake things up*, *others don’t”* [Researcher]
	Requesting (gathering) information	Access is limited, permission has to be requested, and there are delays or failures to reply. Collecting data in the health services requires a lot of management time. *“We would have liked to automate it all [the monitoring]*. *We asked the [national health sector] for permission to link up directly with some of its systems*, *but this never happened*.*”* [Coordinator, international body]
	Ministry staff rotation	Changes in Ministry staff entail loss of learning from monitoring or the need to go through the same procedures again. *“In the end we managed to do all the red-tape*, *but if there’s a change of government [you have to do] everything over again*, *so it’s not efficient”* [Researcher]
Decision-making	Implementation	Maternal health care inequalities are not monitored. *“No doubt this [health care inequities] is a major issue*, *but we haven’t decided how to measure it using any specific indicator”* [Observatory coordinator]
	Monitoring culture	The data are only taken into account for drafting reports; they are not analyzed or used for decision-making. *“The data are used more for reports*, *less for action*. *This is a serious problem because huge quantities of data are generated which are used only for report writing”* [Technician, international body]
	Relations with research	Discrepancies between the time researchers need to generate evidence and the time decision-makers need to make decisions. *“The thing about the decision-makers is that their time-cycles are very short*, *whereas in research we can spend two years looking for resources and then three more carrying it out*, *but no politician is interested in five years”* [Researcher]

Source: Authors’ analysis of responses to semi-structured interview questions with key informants involved in the monitoring of maternal health care in Mexico, 2018.

**Table 3:** Themes and sub-themes identified as opportunities for maternal health care monitoring.

Themes	Sub-themes	Description and example quote
Evaluation	Continuous care perspective	Analyzing continuity of care from pregnancy to postpartum allows the biggest coverage-related challenges to be identified. *“This perspective has been of great use to us for identifying the main challenges for the health service in terms of enhancing coverage”* [Researcher]
	Improve existing indicators	Improving indicators assessed by health systems. Creating tools to facilitate their evaluation and monitoring. *“Some of those indicators already existed in the national systems and some monitoring was performed*. *What we did was to verify the way they were being built*, *if it was appropriate*, *and also generate a way to make it easier to use and analyze a particular item*.*”* [Coordinator, international body]
Information	Diversity of data	A great amount of information is gathered with the aid of information systems and surveys *“Unlike Latin America*, *Mexico has impressive*, *open- access data bases*. *We have data bases for maternal mortality*, *hospital discharges*, *[which may be] good*, *bad or indifferent*, *but many countries don’t have this”* [Researcher]
	Collection	Researcher participation in field work improves data quality. Counting on personnel who visit the services on a regular basis facilitates information gathering. *“Our monitoring was of a very high quality because the researchers did the field work themselves*. *When you delegate or outsource this*, *it’s a disaster*.*”* [Researcher]
Resources	Adequate task force	Including competent collaborators or people involved in the matter helps in performing monitoring. *“Forming a cohesive team uniting the different strengths of each member of the research group enables progress to be made*. *No extraordinary abilities are needed*, *just an interest in the subject”* [Researcher]
Management	Building trust	Building relationships of trust with authorities, decision-makers or peers makes work easier. *“Thanks to [the institution`s] own efforts*, *it has gained a reputation as an institution which people respect for the information it generates*. *So the work itself makes doors open*, *because when your work is being recognized you are granted the confidence to do things*.*”* [Technician, observatory]
Decision-making	Quality indicators	Evaluating quality indicators allows initiatives to be introduced to improve care. *“The ministry has found the quality indicators to be of great use*. *For example*, *they can see which medicaments were lacking or which procedures were followed and which were not”* [Technician, international body]
	Political will	The support and commitment of certain decision-makers facilitates the monitoring of activities or the creation of dedicated organizations. *“A key factor has been the interest shown by the various institutions; being able to rely on the active participation of the institutions not out of any sense of obligation but because they are convinced that this is a priority issue for the country has been fundamental”* [Coordinator, observatory]
	International agenda	Extra impetus is given to the introduction of monitoring systems by the common agenda of international bodies and the health sector. *“It was as part of a cooperative effort between [the international body] and Mexico*, *one of the issues or lines of action was the equity issue”* [Coordinator, observatory]

Source: Authors’ analysis of responses to semi-structured interview questions with key informants involved in the monitoring of maternal health care in Mexico, 2018

## Results

### Monitoring activities

**Fig 2:**
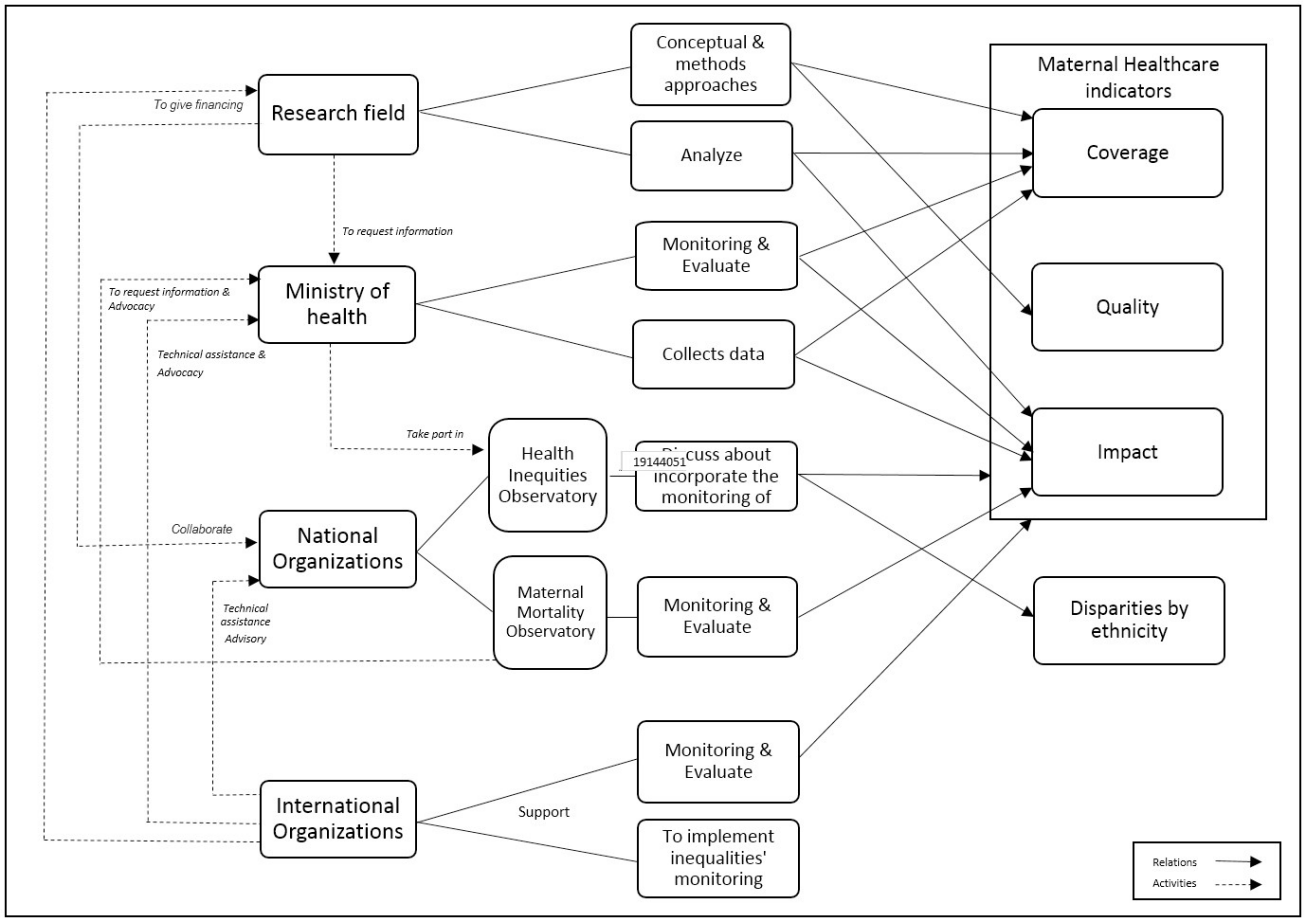
Monitoring of maternal health in Mexico. Source: Analysis of the authors of the documentary review and the responses of the semi-structured interviews questions to key actors.

According to the respondents, the monitoring of maternal health care is performed by measuring or monitoring indicators related to service coverage or quality. Impact measures such as maternal mortality or public policies aiming at its reduction are evaluated (Fig 2).

At all levels (health sector, research, national and international bodies) efforts are made to carry out monitoring, but they are poorly structured and coordinated. Although there are some collaborations between different actors, it is mainly about obtaining information, but in the end, they all work in isolation. The findings or recommendations of national or international research organizations are not considered to improve ministry monitoring. Bureaucracy and lack of communication are the main limitations.

The activities mentioned by the respondents are consistent with the field of action in which they are performed. The health sector gathers data about care through its information systems. Service coverage for pre-natal care, childbirth and use of contraception is measured and monitored. Researchers suggest new ways of monitoring care, analyze gaps between different groups and look for approaches to evaluating service quality. Their principal sources of information are surveys or field work in health services.

Some international organizations work to support the health sector institutions by providing technical assistance to analyze inequalities in health. They have also been designed strategies to implement inequality’s monitoring, but none has yet been implemented in Mexico. Others conduct local fact-finding missions about service quality and measured relevant indicators to monitoring maternal health.

The observatories are directly related to the health sector or have established ties with it; they also collaborate with researchers and international organizations. Their activities are mainly centered on maternal health and health equity, and they attempt to use their evaluations to influence policy-making in their fields of competence. At the time of our study, none of the observatories monitored any indicator related to inequalities in maternal health care.

### Obstacles and opportunities

#### Evaluation obstacles: Aggregate data and complex methods

One limitation pointed out mainly by researchers is the impossibility of carrying out the analysis by subgroups since the data in health sector information systems are generally aggregate. In addition, the system does not organize the records by name, as one researcher remarked: “*there is no way of monitoring these women given the fragmented state of the system*. *If the goal of pre-natal care is to improve health outcomes for mother and child at birth*, *then we need to link the information from the two sources*, *but it can’t be done with the administrative data*.*”*

Researchers, health sector and international organizations mentioned that the methodological approach is a complex task, due in part to the information available, but also to the care-related processes.”*To measure family planning or in-hospital childbirth coverage there are some proxies which let you get a rough idea of what you want to know*, *but sometimes you get a good picture*, *sometimes you don’t*,*”* commented one coordinator.

#### Information obstacles: Poor data quality, representativeness and updating

The main difficulties identified by the respondents regardless of their profile or area of activity are related to data sources. Regarding the information generated by the health sector, as one technician mentioned: “*The problem is data quality*. *When looking for data*, *I come across all kinds of error*, *although it’s not so frequent now*.” Shortcomings were also mentioned related to data collection and irregular reporting intervals. In connection with surveys, researchers pointed out that they are not usually representative locally or for specific groups like the indigenous population. Moreover, there are no guarantees that the information collected in each round will always be the same or that some will not cease to be collected.

Respondents from the health sector and international organizations mentioned that evaluations were not updated due to a delay in the availability of data from the information systems. This fact might be related to the difficulties encountered in implementing a new information system since, as one coordinator commented: “*Not all regions have started using the new system*, *[so] we only have a quarter of the target group*.*”* Respondents showed some dissatisfaction with the lack of up-to-date data, as well as with the new system and how it might modify future evaluations.

#### Resource obstacles: Insufficient funding and personnel for monitoring

Lack of funding is one limitation mentioned by researchers and observatories alike. Researchers point out how difficult it is to obtain funds for projects related to new methods for health care or equity evaluation. As the observatories are not budgeted, they depend on the financing that their members can obtain or on the collaborative work of their participants. As one coordinator put it: “*The collaboration of the institutions is very important*, *we have no budget or staff allocated specifically for the [body’s] activities*. *If support from the various institutions cannot be relied on*, *it’s difficult to get results*.*”* This situation could also be related to the shortage of staff or collaborators to perform monitoring. The health sector and international organizations also refer to staff shortages. Another issue identified in the health sector is overwork since other activities have to be attended to as well as measuring or monitoring the indicators.

#### Management obstacles: Too much bureaucracy, difficult data access and frequent turnover of ministry of health staff

International organizations, observatories, and researchers agree that the bureaucracy hampers decision-making or slows down procedures for doing the work of monitoring. One decision-maker commented: “*There’s been more interest in looking at the bureaucratic aspects [regarding the composition of this body] than in just what is going to be evaluated*.”

At all levels and sectors, the respondents agree that access to some of the data held on information systems is limited. Permission has to be requested, or some personal contact in the relevant unit is needed to facilitate access. Other difficulties cited include delays in receiving the information or failure to reply. Researchers pointed out that a good deal of time is spent dealing with the authorities before information can be collected in the health services.

Directly and indirectly, staff turnover at the Ministry of Health is mentioned and how this affects the continuity of the monitoring process. One technician remarked: “*The high rate of staff rotation at all levels affects us*. *A person learns how to use the data and to make decisions with them*, *but then leaves the institution or is moved to another position*, *and it’s a great loss*”.

#### Decision-making obstacles: Lack of commitment and little familiarity with the use of monitoring

Observatories, international organizations, and researchers acknowledged that the health sector had shown interest in improving monitoring of maternal health care or in measuring its inequalities, but it has not been implemented. One technician said: “*I showed the document and there were some positive comments*, *but nothing came of it*”. Along the same lines a researcher observed: “*We showed it once or twice*, *and the manager was delighted*, *but it got no further*. *It hasn’t had the impact necessary for one of these indicators to be used for the monitoring*.”

International organizations mention that they have noticed little appreciation of the utility of monitoring. The data are usually only taken into account for writing reports, while sometimes they are not analyzed or employed for decision-making purposes.

Another challenge is the discrepancy between the time needed to generate research evidence and the time taken by decision-makers to generate actions; also, achieving fruitful communication between researchers and decision-makers.

#### Evaluation opportunities: Using the continuum of care perspective and improving the indicators

Researchers recommend looking at care as a continuum from pregnancy to postpartum: “*This perspective has been of great use to us for identifying the main challenges for the health service in terms of enhancing coverage*.” International organizations point out another opportunity, namely, to turn again to the indicators the health services already generate to improve them or to create tools to facilitate their evaluation and monitoring.

#### Information opportunities: The great amount of data, involvement in collection, and resource optimization

Researchers believe that despite the limitations regarding quality and opportunity, a considerable amount of data are collected in Mexico: “*Obviously*, *the data aren’t ideal*, *but we have much information which allows us to carry out the analyses*.” In the event of having to collect information themselves, their participation in the fieldwork leads to improved data quality.

On the other hand, international organizations rely on the supervisory teams that regularly visit health services when they want to collect information about the quality of care.

#### Resource opportunities: The need to involve appropriate task forces

Researchers and international organizations acknowledge that being able to count on a team with appropriate training and skills or already involved in the issue allows the research and the monitoring to get done and actions to be generated. One decision-maker remarked: “*We asked the ministries to take on people from various fields*, *not only from maternal and child care*, *[and] we aim to include everyone involved in delivering services with a view to achieving a more systemic view*.*”*

#### Management opportunities: Building stable, inter-sectorial relationships of trust

Health sector and national and international organizations state that building relationships of trust, whether with the authorities, decision-makers or peers within the same institution, improves proceedings at the different stages of monitoring. “*What has helped us to unclog issues of information or red-tape are public relations*, *they’re so helpful*,” one coordinator remarked.

#### Decision-making opportunities: To have quality indicators, political decision-making, and support from the international agenda

One important finding to emerge from international organizations is that the health ministry “*has been finding the quality indicators to be of great use*. *For example*, *they can see which medicaments were lacking or which procedures were followed and which not*”, as one technician noted. So, including quality indicators in monitoring has allowed generating actions to improve maternal health care.

Most respondents agreed that being able to count on the support and commitment of decision-makers allows monitoring to be done correctly or to set up bodies designed to do it. It was possible to use the interviews to confirm that the agenda drawn up between international organizations and the health sector gives added drive to monitoring. Some coordinators commented: “*At the start*, *it was an initiative of an [international body]*, *the health sector and civilian society*,” “*it was as part of a cooperation scheme between the [international body] and Mexico*, *and that’s how it started*.”

## Discussion

### Main findings

This study shows an overview of how the monitoring of maternal health care is carried out in Mexico and the factors that influence the process. Our findings identify the elements that may interfere or facilitate the implementation of the monitoring of inequalities, particularly, by ethnicity.

Our study shows that, in Mexico, although there is particular sensitivity about the importance of measuring inequalities (it has institutions and personnel with experience in monitoring, technical and political support from international organizations) in practice, maternal care inequalities are not measured. We observed that, currently, none of the actors is sufficiently coordinated to do it effectively. There is a lack of commitment and leadership resulting in a set of isolated efforts.

The study unveiled five main categories of obstacles and opportunities related to evaluation, information, resources, management, and decision-making. The main obstacles are related to complex methodological approach, the low quality’s system information, lack of resources and making decisions. Political and technical support from the international agencies to the health sector were the main facilitators.

Recently, in the framework of the SDGs, there has been a discussion of which factors might restrict the performance of health inequality monitoring. The barriers identified emerge from limitations in health sector information systems as well as with political, financial, social and cultural factors. While the key opportunities that countries should move forward include robust data collection infrastructure supported by national institutions, development of technical capacity for equity analysis and communication, and find effective ways to use the results of health inequality monitoring. [7,29]Some of these factors coincide with those found in our study from the participants’ own experience in the monitoring.

The main challenges identified by those who responded referred to information, which has problems of limited access, data quality or inadequate representativeness of surveys in respect of particular interest groups. However, these obstacles are not new for Mexico or for other countries, where for many years it has been pointed out that the information systems are poorly structured, poor data quality due to an inadequate collection, and lack of resources to conduct more extensive or specific surveys. [30–32] Address these problems are essential in order to obtain better evaluations to better guidance for decision-making.

In recent years, health inequalities monitoring has gained ground in the political attention of the countries [7,33–35] Our results show that key professional at different levels such as technicians, coordinators, researchers or decision-makers are not only aware of the issue but also of its importance. However, this does not lead to political decisions to develop any measure for monitoring maternal health care inequalities in Mexico. This fact could be related to the lack of familiarity with the use of monitoring for decision making. [7,30,35] Therefore, we thought that it is essential to stress the need to establish some institutional link between the results of monitoring and concrete actions that might reduce the inequalities revealed in the monitoring.

As for opportunities, our findings suggest that the role of international organizations and their links with the countries is crucial to carry out monitoring. In this connection, given the international commitment “not to leave anyone behind,” many international organizations and initiatives are providing technical support in metric generation, enhancing monitoring systems and strengthening capacities to analyze results and put them to good use. [7,33,34] Provided that full advantage is taken of the support, cooperation and political influence of this types of organizations or initiatives, progress will be made in implementing health inequality monitoring.

### Policy implications

Nobody doubts the need for ethnicity-based monitoring of maternal health care inequities if it is to be reduced. Monitoring would enable differences concerning other groups to be identified, as well as the critical points of care coverage. [36,37] However, our study shows that although there is some sensitivity towards measuring inequalities, in practice, there is still no indicator to monitor maternal health disparities.

Monitoring could play a strategic role by making decision-making more efficient and effective through the provision of information about whether policies, schemes or activities are accomplishing the purpose for which they were set up or designed. [5,12] The government, the health ministry, and other decision-makers could focus research on those areas in order to determine the cause of the problems and improve the state of maternal health among the indigenous populations.

Given our findings, we believe that in the short-term surveys might be used as the most feasible source of information, due to the poor quality, delays or lack of data available in the health sector information system. In the future, once the Mexican system is fully operational, it could be reconsidered as a possible resource.

As for evaluations, the evidence generated from researchers or deriving from the experiences of international organizations should be reviewed and analyzed to identify the most feasible and useful indicators for decision-making at the national and regional levels. According to our study, there are enough proposals that could be taken up by the observatories or the ministry of health.

Overcoming the challenges related to management and decision making for the implementation of monitoring by ethnicity requires that some of the actors involved take the commitment and leadership. The institution or area concerned should articulate and coordinate the efforts being made from different institutions. In the case of Mexico, we thought that the collaboration of the observatories could be essential to propose the implementation of monitoring inequalities in maternal healthcare. They would exchange their knowledge and experience in monitoring maternal health and how to measure disparities in health.

The political and technical support being lent currently to this issue by international organizations is an opportunity for placing ethnicity-based monitoring of maternal health care on the decision-makers’ agendas. In the past, Mexico has taken advantage of this drive to advance in health monitoring, for example, with the setting up of monitoring organizations. However, care must be taken that monitoring is continuous and mechanisms need to be defined to guarantee its future regardless of international support or changes in the administration.

## Limitations

Our use of a qualitative method means that our findings derive from our respondents’ experiences and cannot, therefore, be generalized. One of the main limitations is that only one of the public health service providers was included in the study, albeit the main provider of services to groups at risk of suffering inequalities in health care as well as the provider responsible for the Mexican health sector. It is, therefore, possible that the obstacles and opportunities for other institutions may differ from those that emerge in this study; that said, having achieved discursive saturation; we have obtained the most significant opinions in this field of activity.

## Conclusion

As we have argued, the success of a monitoring system to help decision-making aimed at reducing maternal health inequalities seems to depend not only on accurate evaluation but also on the context in which it is carried out and the actors involved, which is why it is fundamental to take their perspectives into account.

Our study has permitted us to analyze the case of maternal health care in Mexico as it relates to the implementation of ethnicity-based inequality monitoring. Nevertheless, actors from other countries may identify themselves with the views of our respondents and find in them a precedent demonstrating the need to understand the workings, obstacles, and opportunities for monitoring as they currently perform it; thus, progress may be made towards assessing inequalities and thereby reducing health inequities with the aid of concrete policies and initiatives.

## Supporting information

S1 Table Participants’ characteristics. ^a^Mean; ^b^Experience in general monitoring activities not necessarily in maternal health. Note: All actors of the Ministry interviewed was from the national level. Sources: Own elaboration using information from the questionnaire applied to participants.
(PDF)
