# Supplementary material for: Obstacles and opportunities for monitoring ethnicity-based inequalities in maternal health care: Lessons from Mexico
Source: PLoS One. 2019 May 31;14(5):e0217557. doi: 10.1371/journal.pone.0217557 (PMC6544348; doi:10.1371/journal.pone.0217557)
Supplement: S1 Table — aMean; bExperience in general monitoring activities not necessarily in maternal health. Note: All actors of the Ministry interviewed was from the national level. Sources: Own elaboration using information from the questionnaire applied to participants. (PDF) [file pone.0217557.s001.pdf]

S1 Table. Participants' characteristics

**General**	
No. interviews	17
Gender	
Female	59%
Male	41%
Education level	Postgraduate
**By profile**	
Technicians	4
Age[a]	36 years
Experience in monitoring[ab]	5 years
Institutions	Ministry of Health<br>National organizations<br>International organizations
Activities	Estimation, monitoring, technical assistance, research
Coordinators	4
Age	45 years
Experience in monitoring	6-10 years
Institutions	Ministry of Health<br>National organizations<br>International organizations
Activities	Linking, management, monitoring
Decision- makers	4
Age	48 years
Experience in monitoring	More than 10 years
Institutions	Ministry of Health<br>National organizations<br>International organizations
Activities	Advocacy in public health, evaluation, research
Researchers	5
Age	49 years
Experience in monitoring	More than 10 years
Institutions	Academic institutions
Activities	Teaching and research

[a] Mean; [b] Experience in general monitoring activities not necessarily in maternal health
Note: All actors of the Ministry interviewed was from the national level.
Sources: Own elaboration using information from the author's questionnaire applied to participants
